# A Mathematical Model of Average Dynamics in a Stem Cell Hierarchy Suggests the Combinatorial Targeting of Cancer Stem Cells and Progenitor Cells as a Potential Strategy against Tumor Growth

**DOI:** 10.3390/cancers12092590

**Published:** 2020-09-11

**Authors:** Rodolfo Molina-Peña, Juan Carlos Tudon-Martinez, Osvaldo Aquines-Gutiérrez

**Affiliations:** School of Engineering and Technologies, Universidad de Monterrey, San Pedro Garza García, NL 66238, Mexico or rodompe@gmail.com (R.M.-P.); osvaldo.aquines@udem.edu (O.A.-G.)

**Keywords:** CSC targeting, tumor relapse, hierarchical mathematical model, minimal model, progenitor cells, combination therapy

## Abstract

**Simple Summary:**

Cancer stem cell (CSC) directed therapies have been increasingly developed during the last years. However, some reported experiments using this strategy showed that although delayed tumor growth was observed, the tumor was not completely eliminated. Here, we hypothesize that the simultaneous targeting of CSCs and progenitor cells of intermediate phenotype may represent a better strategy against tumor growth. We aimed to fit a mathematical model, consistent with the CSC hypothesis, to reported experimental data resulting from CSC direct targeting. This is a minimal model of average tumor dynamics that could aid in the visualization of the overall tumor growth when different subpopulations of tumor cells are targeted. We show that combination therapy during a time lapse that ensures eradication of CSCs and progenitor cells in a stem cell hierarchy controlled tumor relapse. Testing this hypothesis in vivo may help to discriminate among other possibilities of tumor burden.

**Abstract:**

The cancer stem cell hypothesis states that tumors are maintained by a small subpopulation of stem-like cells, often called cancer stem cells (CSCs) or tumor initiating cells. CSCs can self-renew and give rise to more differentiated cells, which comprise the bulk of the tumor. In addition, CSCs are resistant to conventional therapy, which suggests that they are responsible for tumor relapse. This has led researchers to increase efforts to develop directed therapies against CSCs. However, some experiments in mice have shown that the elimination of CSCs might not ensure tumor eradication. This may be due to different events, such as residual CSCs after treatment, the plasticity of cells within the tumor, the presence of different CSCs having their own hierarchy within the same tumor, and the ability of more differentiated cells to maintain the disease, among others. Trying to decipher this complexity may benefit from dissecting the whole in its parts. Here, we hypothesize that tumor relapse after the selective targeting of CSCs may be due to intermediate progenitor (P) cells that can maintain the tumor volume. In order to support the hypothesis, we implemented a mathematical model derived using pseudo-reactions representing the events of each cell subpopulation within the tumor. We aimed to test if a minimal unidirectional hierarchical model consisting of CSCs, P, and terminally differentiated (D) cells could be adjusted to experimental data for selective CSC targeting. We further evaluated therapies ranging from nonselective to specifically directed and combination therapy. We found that selective killing of the CSC compartment has a delaying effect on the overall exponential tumor growth, but was not able to eliminate the disease. We show that therapy that targets both CSCs and intermediate progenitor (P) cells with a sufficient capacity to proliferate and differentiate could represent a more efficient treatment option for tumor depletion. Testing this hypothesis in vivo may allow us to discriminate within the array of possibilities of tumor relapse, and further open the idea of combination therapy against different subpopulations of tumor cells instead of segregating CSCs and bulk tumor cells.

## 1. Introduction

The most fundamental trait of cancer cells is their sustained and uncontrolled proliferation due to the acquisition of somatic mutations and dysregulation of signals that normally control cell growth [1]. Two theories can explain the origin of cancer cells. The stochastic model of cancer suggests that tumors may originate from spontaneous mutations in random cells attributing them their tumorigenic properties [2,3]. In contrast, the cancer stem cell hypothesis states that tumors are constituted as a hierarchy with cancer stem cells (CSCs) at the apex of the branching process [3,4]. It is believed that the origin of CSCs is normal stem or progenitor cells due to their long living capacity and hence, their greater susceptibility to accumulating mutations [5].

In agreement with the CSC hypothesis, numerous studies have reported the existence of CSCs in leukemia and solid tumors [6]. CSCs constitute a minor fraction of the tumor, typically between 0.1% and 2.5%, and are usually sorted by the expression of specific cell surface markers or enzymes [3,6,7]. The fundamental characteristic of CSCs is that they can reconstitute the tumor when transplanted into immunodeficient mice, with the restoration of heterogeneity presenting different degrees of cell differentiation. For instance, Collins et al. [8] isolated CSCs, transit amplifying cells, and differentiated cells from prostate cancer. In their experiments, CSCs demonstrated a greater colony-forming efficiency and could give rise to the other subpopulations of differentiated cells in vitro. Similarly, Patrawala et al. demonstrated this phenomenon in vivo in prostate [9] and breast [10] cancers. Furthermore, they proposed a hierarchical model to explain the differential phenotypic expression and tumorigenic capacity of the different cell subpopulations.

Another characteristic of CSCs is their resistance to conventional therapies, e.g., chemotherapy and radiation [11]. This raises the possibility that CSCs are responsible for tumor relapse. Because of the tumor seeding properties and resistance of CSCs, there is an ongoing effort to develop selective therapies against CSCs, as is evident from patents (e.g., US Patent 9023338) [12] and scientific publications [7]. For example, Gupta et al. [13] found that salinomycin, which is an ionophore compound, selectively killed breast CSCs, and Zielske et al. [14] were able to selectively ablate CSCs with radiation. However, in both studies, the experimental growth curves indicated relapse after treatment, showing that the treatment was not totally efficient and only resulted in a delaying effect.

Possible explanations for these phenomena are (a) residual CSCs that were not efficiently eliminated when specifically targeted; (b) the plasticity observed in more differentiated cells, as evidenced by reprograming to a stem-like state when exposed to transcription factors that induce epithelial-to-mesenchymal transition (EMT) [15]; (c) the coexistence of different CSC populations within the same tumor, as recently suggested [16]; and (d) the presence of more differentiated progenitor cells capable of maintaining the tumor.

Many biological aspects of CSCs in solid tumors remain to be further investigated. For example, while some authors consider that CSC features and EMT processes are associated [15], others suggest that EMT is not always applicable [17,18]. Moreover, some researchers have reported that EMT is a requisite for metastasis [19], whereas others have found that this could be dispensable [20,21]. Furthermore, whether different populations of reported CSCs within the same tumor belong to completely different lineages or the same hierarchy, with one being more primitive than the other, has not been fully elucidated.

Recent findings by Lan et al. in terms of glioblastoma multiforme tumors have evidenced an invariable hierarchy showing cells with different proliferative capacities, including CSCs, intermediate progenitors, and differentiated cells [22]. The authors highlighted that exponential growth could not only be explained by cancer stem cell divisions. In another study, Koh et al. observed a differential expression of embryonic stem cell markers in head and neck cutaneous squamous cell carcinoma [23]. The authors speculated about a hierarchy of a more primitive stem-like cell expressing OCT-4(+)Nanog(−) and a more differentiated progenitor corresponding to an OCT-4(+)Nanog(+) phenotype.

Typically, papers describing CSC characterization by implantation in immunodeficient mice compare the grafting efficiency as relative to the non-CSC fraction, concluding an x-fold increment in tumorigenicity for the CSC fraction. However, in some cases [9,10,24,25,26,27,28], the non-CSC fraction, although reduced, also shows a tumorigenic capacity. This raises the possibility that intermediate progenitor cells that may be contained within the non-CSC fraction are indeed able to form tumors or sustain tumor growth once created. This suggests that a lineal hierarchical model is still relevant to study during tumor development.

Here, we hypothesize that intermediate progenitor (P) cells are necessary for tumor growth and that the relapse observed in CSC targeting approaches can be explained by the presence of more differentiated progenitors that are still capable of proliferation. In order to support the hypothesis, we used a pseudochemical mathematical model in which each event of cell division and cell death is represented as a pseudo-chemical reaction mediated by a kinetic constant *k_j_* that represents the rate at which each cellular event takes place.

In this paper, we fit the mathematical model to experimental tumor growth curves for the selective targeting of CSCs and show that the model fits well, reproducing the fractions of CSCs at the final experimental point, underpinning that tumor relapse can be explained by the presence of intermediate P cells. Next, we theorize an effective treatment for tumor eradication by using combination therapy that targets both CSCs and P cells. Although a minimal model without plasticity was implemented, it allowed us to explain that the design of CSC-directed therapies should also consider targeting of the intermediate compartment in a hierarchical model. Finally, we conclude with a discussion of the model limitations and a sketch of an experimental model to verify the hypothesis.

## 2. Methods

### 2.1. Hypothesis

The tumor burden after selective treatment against CSCs may be due to the following:

1. Residual CSCs;
2. More differentiated cells regaining a CSC ability;
3. Different CSC populations within the same tumor;
4. Intermediate progenitor (P) cells that possess enough potency to generate tumors.

Firstly, there is the possibility that CSC direct targeting does not reach all CSCs, and hence, residual CSCs are able to regrow and repopulate the tumor. This could be due to the administration of an inadequate treatment dose or the inability of the therapeutics to reach the target. Here, we suppose that specific cell targeting can reach efficiency at a hypothetical dose of the therapeutics and treatment duration in the different scenarios simulated. By doing so, we can vary the dose and treatment time and observe the behavior of the different cell subpopulations during the course of treatment.

Secondly, another possibility is that more differentiated cells regain stem-cell-like features. Indeed, Mani et al. reported that by inducing EMT by transducing Twist or Snail transcription factors in an immortalized epithelial breast cancer cell line, modified cells were increased in the number of CD44^high^CD24^low^ phenotypes with CSC characteristics [15]. The authors hypothesized that EMT could explain macroscopic metastasis. In the same line, Tsai et al. found that reversible EMT was required for metastasis to occur in a squamous cell carcinoma model [19]. However, further on, it was demonstrated that EMT is dispensable for the metastasis of lung and pancreatic cancers [20,21]. More recently, Fumagalli et al. investigated the role of Lgr5(+)-CSCs and their negative counterpart in colorectal cancer metastasis [29]. They showed that more differentiated Lgr5(−)-non-CSCs were able to metastasize and form secondary tumors by dedifferentiation to Lgr5(+)-CSCs. Lgr5(−) cells were more invasive and circulated in the blood with a greater frequency than Lgr5(+)-CSCs. This suggests that EMT was not necessary for the initial steps of metastasis, but plasticity of the non-stem cell fraction was fundamental for reconstitution of the secondary tumor.

Although these research efforts reveal that cell plasticity can be involved in the process of metastasis, its role in the case of primary tumor growth dynamics is less clear. Pathologists have mentioned that in histological sections of human pancreatic cancer, it is rare to observe cells with a mesenchymal morphology [30], and recent evidence shows that the deletion of EMT does not alter primary tumor growth and local invasiveness [21]. More investigations are needed to gain further insights into this phenomenon. In the present work, we did not consider plasticity, mainly because we focused on a minimal model for primary tumor growth with the aim of assessing whether this could explain tumor relapse.

Thirdly, the existence of different populations of CSCs within the same tumor could also explain tumor relapse. The direct targeting of one CSC population while leaving the other unaffected could lead to a new established hierarchy by the latter that would direct the tumor burden. This idea has been put forward elsewhere [16]. Although recent reports [31,32,33,34,35] mention the existence of different populations of cancer cells differentially expressing embryonal stem cell markers at the mRNA and protein level, confirmation of their stemness potential and whether they belong to the same hierarchical structure were not tested. In addition to this complexity, Liu et al. postulated that CSCs can shift between two different stem-like states [36]. They showed that two CSC populations exist in breast cancer: One CD44(+)CD24(−)-slow-proliferative-mesenchymal-like CSC and an ALDH1(+)-high-proliferative-luminal-like CSC. After the isolation of each one, they could independently reconstitute the heterogeneity initially observed. However, little attention was paid to the small overlapping population CD44(+)CD24(−)ALDH(+) cells, raising the possibility that, again, a lineal hierarchy could be involved. This could probably be assessed by including a reporter in a construct that allows researchers to determine if genes involved in EMT and its reversal—mesenchymal-to-epithelial transition (MET)—are activated. The question of whether there are different CSC populations within a tumor remains open, and here, we do not consider this complexity, in order to determine whether a minimal model can explain the tumor burden after the selective targeting of a unique population of CSCs.

Finally, a fourth possibility is that intermediate progenitor (P) cells that are created by CSCs have enough potency to generate and maintain tumors. Typically, the evaluation of stemness is assessed by the ability of sorted cell fractions to form tumors in immunodeficient mice. Despite the fact that the stem-cell-like fraction usually has an x-fold greater tumorigenic capacity compared to the non-stem-cell counterpart, the latter fraction, in some cases, also shows a reduced, but still latent, capacity to form tumors (see, for example, references [9,10,24,25,26,27,28]).

This observation has been noted before by Kern and Shibata, who, with a ponderation analysis, showed that the non-stem cell fraction has a tumorigenic index possibly due to contamination with CSCs or the defined stem cell markers being too restrictive [37]. One way to avoid contamination with other cell types is to use clonal assays. By doing so, Kim et al. showed that more differentiated breast cancer luminal-like cells had the capacity to form clones that remained luminal, whereas the basal stem-like cells could form clones with a reconstituted heterogeneity containing luminal cells [17]. In addition, luminal cells had the capacity to form tumors in vivo that were even more invasive than the tumors originating from the basal stem-like cell fraction, despite the fact that they did not observe evidence of plasticity. Furthermore, as described before in their clonal assay, only the tumors formed by the basal stem-like fraction were reconstituted as heterogeneous, whereas tumors from more differentiated cells remained luminal. In another study, an elegant graphical analysis by Patrawala et al. showed that the postulated prostate cancer intermediate progenitor cells formed xenograft tumors ([9], their Figure 4). In a more recent report, in glioblastoma multiforme, progenitor cells were essential for explaining exponential growth in a conserved hierarchical model that determined a linear cell hierarchy by using DNA barcoding analysis [22].

This last presented evidence suggests that even if the non-stem cell fraction is more differentiated, proliferation and tumorigenicity within it is still possible. If, for example, P cells do not exhibit extensive proliferation, but only 10 rounds of cell division, the number of produced cells will be P_initial_ × 2^10^. Then, if we suppose P_initial_ = 3.2 × 10^5^ cells, and the tumor density is constant and equal to 3.2 × 10^5^ cells/mm^3^ (see the section below), the new volume will be 1 mm^3^ × 2^10^ = 1024 mm^3^ ~ 1 mL, which becomes a palpable tumor. Therefore, P cells might be an important subpopulation to consider when designing CSC-directed strategies.

Despite the fact that all four points discussed above deserve to be considered as a potential source of tumor relapse, trying to decipher this complexity may benefit from dissecting the whole in its parts. Based on the experimental evidence discussed in point four, we hypothesize here that, in a hierarchical minimal cellular model within the tumor, even after ablation of the CSC compartment and supposing that there are no state transitions or that they are in equilibrium, i.e., the rates of the terminal differentiation of CSCs and de-differentiation of terminally differentiated cells to CSCs are equal, there are sufficient numbers of intermediate progenitor (P) cells such that they can regain exponential growth after a number of rounds of cell division. Therefore, therapy that targets both CSCs and intermediate progenitor (P) cells with a sufficient capacity to proliferate and differentiate could represent a more efficient treatment option for tumor depletion. In order to support this hypothesis, we present the following mathematical model.

### 2.2. Mathematical Model

**Figure 1:**
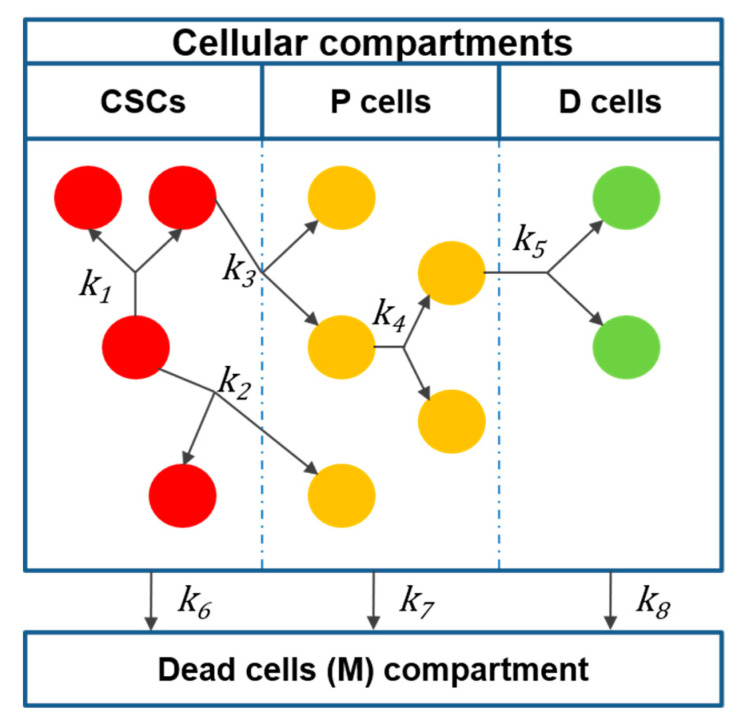
Compartmentalized representation of the proposed model. Each cell event is mediated by a rate constant *k*_j_. Cancer stem cells (CSCs) can self-renew (*k*_1_) or give rise to intermediate progenitor (P) cells through either asymmetric (*k*_2_) or symmetric (*k*_3_) division. P cells, in turn, can proliferate (*k_4_*) and give rise to terminally differentiated (D) cells (*k*_5_). Each cell type has a death rate represented by *k_6_* for CSCs, *k_7_* for P cells, and *k_8_* for D cells.

We will briefly describe our previous mathematical model [38]. We consider the cellular division events presented below and represent them as pseudochemical reactions mediated by rate constants *k_j_* (Figure 1). By doing so, each cellular event can be followed during tumor development.

CSCs can expand in number through symmetric self-renewal [39]:

(R1) $$CSC\overset{\;k_{1}\;}{\to }2CSC.$$

CSCs can also undergo asymmetric division, maintaining their numbers and giving rise to one intermediate progenitor (P) cell [39]:

(R2) $$CSC\overset{\;k_{2}\;}{\to }CSC+P.$$

CSCs can also undergo symmetric differentiation [40,41]:

(R3) $$CSC\overset{\;k_{3}\;}{\to }2P.$$

Intermediate P cells can self-renew by symmetric cell division [42]:

(R4) $$P\overset{\;k_{4}\;}{\to }2P.$$

P cells can also divide and terminally differentiate into D cells, which do not have the capacity to proliferate [42]:

(R5) $$P\overset{\;k_{5}\;}{\to }2D.$$

Each cell subtype can undergo cell death:

(R6) $$CSC\overset{\;k_{6}\;}{\to }M,$$

(R7) $$P\overset{\;k_{7}\;}{\to }M,$$

(R8) $$D\overset{\;k_{8}\;}{\to }M.$$

The model is derived by establishing a cellular balance for each cell subtype: 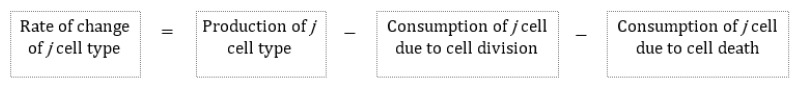


That is mathematically expressed as

(1) $$\frac{dj}{dt}=r_{production,j}-r_{cell\;division,j}-\;r_{cell\;death,j}.$$

Production and consumption rates were calculated by considering each cellular step as an elementary reaction. With the coefficient for each cellular subtype on the left side of each “reaction” being equal to one, the rates will be of the first order, i.e., the rate for a particular cell subtype will depend on the amount of cells of that subpopulation at a particular time. The model has one kinetic parameter per cellular event defined by *k_j_*, for example, *k_1_* is the intrinsic reaction rate constant that indicates the natural tendency of CSCs to divide symmetrically to produce two CSCs (R1). The rates of production and consumption of each cellular event depend on both the number of precursor cells for that event and the constant *k_j_* that will multiply that number. For example, the rate of production of CSCs in R1 will be two-fold (*2k_1_CSC)* its rate of consumption due to cell division (*k_1_CSC)*. By implementing this strategy, the calculations of production and consumption rates for each event where CSCs are involved are as shown in Table 1. The sum of all rates in each column represents the total rate of production (left) and the total rates of consumption due to cell division (center) and cell death (right) for this cell type.

Then, by applying Equation (1), the rate of change for CSCs is given by

(2) $$\frac{dCSC}{dt}=\left(2k_{1}CSC+k_{2}CSC\right)\;-(k_{1}CSC+k_{2}CSC+k_{3}CSC)-k_{6}CSC.$$

Simplifying Equation (2), we can obtain

(3) $$\frac{dCSC}{dt}=\left(k_{1}-k_{3}-k_{6}\right)CSC.$$

Similarly, by applying this strategy for P and D cells, the following differential equations are obtained:

(4) $$\frac{dP}{dt}=\left(k_{2}+2k_{3}\right)CSC+\left(k_{4}-k_{5}-k_{7}\right)P,$$

(5) $$\frac{dD}{dt}=2k_{5}P-k_{8}D.$$

To calculate the total number of living cells, we sum all subpopulations:

(6) N=CSC+P+D.

The tumor volume is then calculated by approximating the contribution of the total number of cells to the tumor volume in mm^3^. The average cellular density was estimated from the literature to be in the order of magnitude of 10^8^ cells/cm^3^ when considering 100–1000 mm^3^ tumors [43,44,45]. This order of magnitude is more appropriate for tumors of epithelial origin [46].

The tumor volume is given by the relation

(7) $$V\left(mm^{3}\right)=N/3.2\times 10^{5},$$

where $3.2\;\times \;10^{5}$ is the average cellular density in cells/mm^3^.

In order to explore the solution of the model, different relationships between specific rate constants were defined by Φ_i/j_ = [Φ_2/1_, Φ_3/1_, Φ_4/1_, Φ_5/4_, Φ_6/1_, Φ_7/1_, Φ_8/1_]. This parameter indicates the proportions of the defined relations between specific rate constants *k_j_*. For example, Φ_2/1_ = *k_2_/k_1_* is the ratio between asymmetrical and symmetrical CSC specific renewal rate constants, corresponding to cellular reactions denoted by R2 and R1, respectively. Similarly, Φ_5/4_ = *k_5_/k_4_* indicates the relative magnitude between the specific rate constants associated with the symmetrical differentiation (R5) and renewal (R4) of progenitor cells.

In our previous report [38], we adjusted this model to three experimental in vivo tumor growth scenarios. We found that tumors may follow common paths of cell division. This is because the parameter space of the model (Φ_i/j_) was maintained for all experimental datasets, i.e., Φ_2/1_ = 1, Φ_3/1_ = 0.01, Φ_4/1_ = 5.35, Φ_5/4_ = 0.8, Φ_6/1_ = 0.01, Φ_7/1_ = 0.1, and Φ_8/1_ = 1. This solution was obtained by varying the value of Φ_i/j_ so that the model fitted the experimental tumor volume points and, at the same time, satisfied the following constraints:

(8) $$k_{6}<\;k_{7},$$

(9) $$k_{7}<\;k_{8},$$

(10) $$d\left(\frac{CSC}{N}\right)/dt=0,$$

(11) P/N≤0.2,

(12) D/N≥0.8.

This solution was chosen because *k* values are more representative of some biological observations. For example, if we assign the value of 1 to *k_1_*, then, *k_2_* = 1, *k_3_* = 0.01, *k_4_* = 5.35, *k_5_* = 4.28, *k_6_* = 0.01, *k_7_* = 0.1, and *k_8_* = 1. The higher values of *k* corresponding to P cell divisions compared to the *k* values related to CSC divisions mean that the frequency of CSC divisions is slower than for P cells, which is in accordance with experimental data [22,27]. Similarly, the rate of cell death of terminally differentiated (D) cells is 10-fold higher than for P cells and 100-fold higher than for CSCs [11]. For further details, please refer to Molina and Alvarez [38].

We used this previous model and parameter space and adjusted it to the new experimental data presented in this report. To validate it, we relied on two experiments: One using radiation against CSCs [14] and the second using salinomycin that selectively killed CSCs [13]. These experiments were selected because the authors reported the fractions of CSCs at the beginning and the end of the experimental points. We explored the selective targeting of CSCs and compared the model output to the experimental data. We then simulated different strategies ranging from nonselective bulk tumor treatment, directed therapy against CSCs and P cells, and combination therapy that could potentially represent a better treatment strategy than CSC targeting alone. Finally, we extrapolated the findings to a real experimental tumor growth curve in which combination therapy was used [47]. For each experimental case, we set the initial number of cells in each compartment, CSCs, P, and D cells, as experimentally reported. When treatment was administered, we increased the cell death rate of the corresponding subpopulation during the time of treatment so that the curve fitted the experimental data points. Similarly, for the simulated combination therapy, we modified the *k_j_* values corresponding to each scenario that we will present in the next section. The model output is presented in figures where the CSC and P fractions are also plotted as means of following the evolution of both cell subpopulations during the treatment.

## 3. Results and Discussion

### 3.1. Tumor Relapse after the Selective Targeting of CSCs

The fact that CSCs make up a minor subpopulation of cells and that intermediate progenitor (P) cells, which are more abundant, also have the ability to proliferate, raises the possibility that even in the case of the effective elimination of the CSC compartment, other cells are able to sustain tumor growth. In this regard, Kern and Shibata [37], in a simple analysis, showed that the non-CSC fraction could have a tumorigenic index, Lan et al. showed that proliferative P cells explain exponential growth in glioblastoma [22], and different studies have reported that more differentiated cells are able to form tumors [9,10,17,25].

Some experiments testing the effect of selective CSC targeting support our hypothesis. For example, Zielske et al. [14] found that the selective ablation of CSCs with radiation had a delaying effect on xenograft growth, and Gupta et al. [13], through high-throughput screening, discovered that salinomycin was selectively toxic to CSCs. In both cases, during the treatment experiments, the tumor relapse curves showed a lag period, after which tumor growth continued with the same profile as the controls.

**Figure 2:**
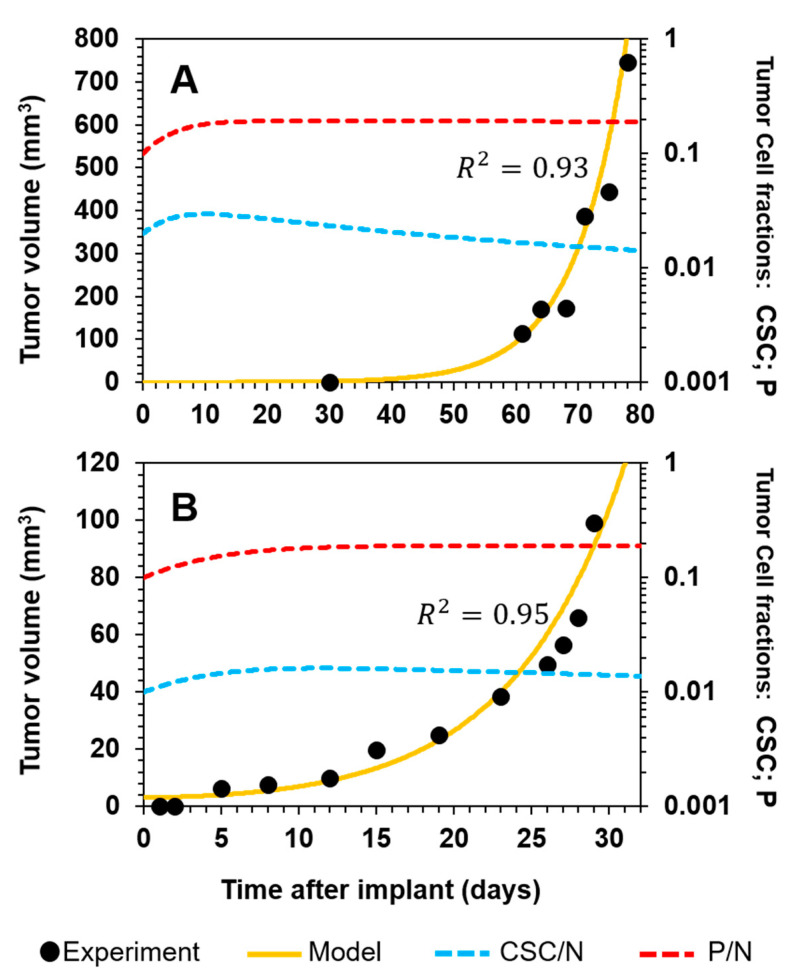
Model fitted to control experiment scenarios. (**A**) Human-derived breast cancer cells (3 × 10^4^) were implanted in the mammary fat pads of mice [14]. (**B**) Human breast cancer cells (1 × 10^6^) were implanted in the inguinal mammary glands of mice [13]. Experimental data are represented by black points, model fitting by the yellow line, the CSC fraction by the dashed blue line, and the P fraction by the dashed red line.

To gain further insight into the dynamics of CSC targeting and tumor relapse, we used our previous model. First, we adjusted the model to control curves (no treatment with either radiation or salinomycin) to verify that the same rate dynamics were followed (i.e., the same Φ_i/j_ relationships previously defined were maintained) and, hence, that the model was applicable to the new experimental scenarios. This was shown to be the case, as the model curves fitted the control experiment data points reported by Zielske et al. [14] (Figure 2A) and Gupta et al. [13] (Figure 2B), with coefficients of determination (*R*^2^) of 0.93 and 0.95, respectively.

**Figure 3:**
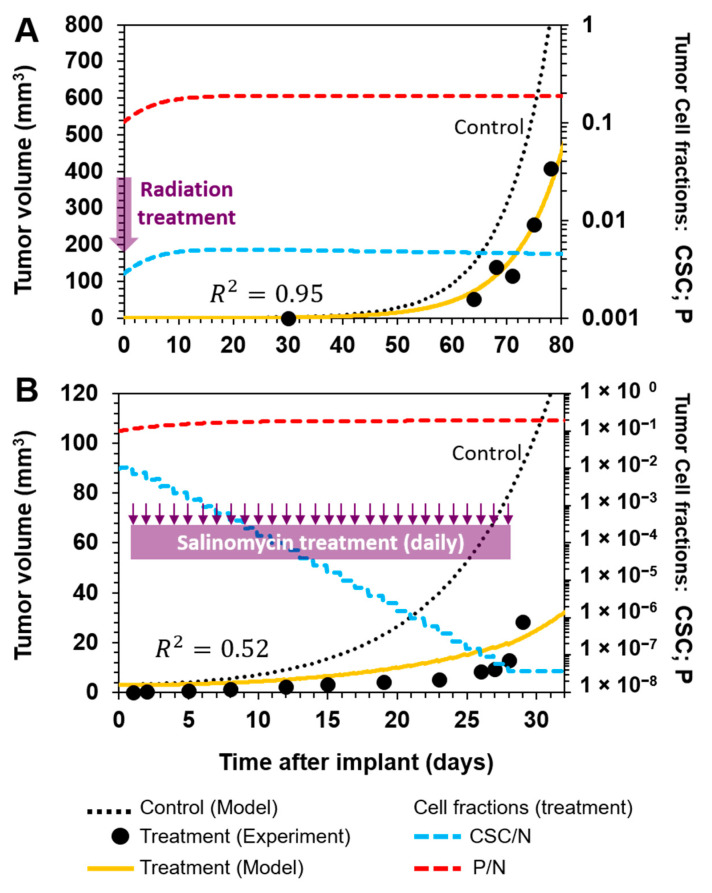
Model fit of experimental treatment scenarios targeting CSCs. (**A**) Radiation-treated cancer cells (3 × 10^4^) were implanted in the mammary fat pads of mice [14]. This treatment reduced the percentage of CSCs in the inoculation culture. Tumor growth was delayed compared to the control, but the tumor relapsed and the fraction of CSCs increased over time to reach a new equilibrium. (**B**) Breast cancer cells (1 × 10^6^) were implanted in the mammary glands of mice, which, after 24 h, were then administered daily with salinomycin for four weeks [13]. Selective elimination of CSCs considerably delayed the tumor burden. However, the tumor regrew, despite the elimination of CSCs. Experimental data are represented by black points, model fitting by a yellow line, the control treatment curve by a dashed black line, the CSC fraction by a dashed blue line, and the P fraction by a dashed red line.

Having verified how the model fitted the control data, we next evaluated the treatment corresponding to each experiment. In the first case, Zielske et al. [14] used a radiation dosage of 8 Gy on human breast tumor cells that were implanted in the mammary fat pads of mice. This treatment reduced the number of CSCs in the inoculation culture from 2% to 0.31%, as assessed by flow cytometry. The results show that, despite substantial improvement being produced through the slowing of the tumor burden, this was only temporary, with growth being delayed for a period of approximately four weeks before subsequently following the same trend as that of the control curve (Figure 3A). When we tested this treatment in the model by only changing the initial conditions (i.e., the number of each cell type, as experimentally determined) while maintaining all other parameters as fixed, we observed that the model fitted (*R*^2^ = 0.95) the experimental data points well (Figure 3A). Furthermore, the model can accurately describe the experimental observations of the increase in the fraction of CSCs, which reached a new equilibrium (Figure 3A, blue dashed line).

In this experiment, tumor relapse might have been due to the contribution of residual CSCs following radiation. Despite the fraction of CSCs being substantially reduced after treatment, this smaller population of CSCs was able to regrow and reach a new equilibrium. Although this experiment supports our hypothesis in a way, it does not demonstrate that tumors can relapse after eradication of the CSC compartment. However, it allowed us to test the validity of our model.

We next moved to the experiment reported by Gupta el al. [13], in which they used salinomycin, which is a compound selectively toxic to CSCs. In contrast to Zielke et al. [14], they allowed the untreated inoculation culture to grow for one day in the inguinal mammary glands of mice, and then initiated treatment by administering 5 mg/kg of salinomycin to animals on a daily basis. Again, although a substantially improved slowing of the tumor burden was observed, tumor relapse occurred after 30 days (Figure 3B). In this case, elimination of the CSC compartment was assessed by determining the expression of E-cadherin, which is an epithelial differentiation marker. After four weeks of treatment, the analysis of the composition indicated that the tumor was primarily composed of cells expressing this marker.

We simulated the daily selective killing of CSCs by augmenting the death rate of this subpopulation during the four-week course of treatment. The results show that the model described the overall behavior of the experimental treatment curve (*R*^2^ = 0.52) and tumor cell composition with the gradual eradication of CSCs (Figure 3B, blue dashed line). This result supports our hypothesis, suggesting that even if CSCs were eliminated, more differentiated cells might be able to continue growing and, hence, allow the tumor to relapse, albeit with a different composition of cell subpopulations.

As these results validated the use of this model as a simple tool for describing the experimental growth of tumor implantation and relapse after treatment, we then used it to evaluate different strategies—nonselective, selective, and combinatorial treatments—as described in the following paragraphs.

### 3.2. Comparing Strategies to Combat Cancer

Using our model and the experimental tumor curve reported by Ricci-Vitiani et al. [48] for colon cancer, we tested different tumor treatment scenarios, namely conventional nonselective therapy and the specific targeting of tumor cell subpopulations, in order to compare the tumor eradication efficiency of the selected strategies.

#### 3.2.1. Nonselective Treatment of Tumor Cells

**Figure 4:**
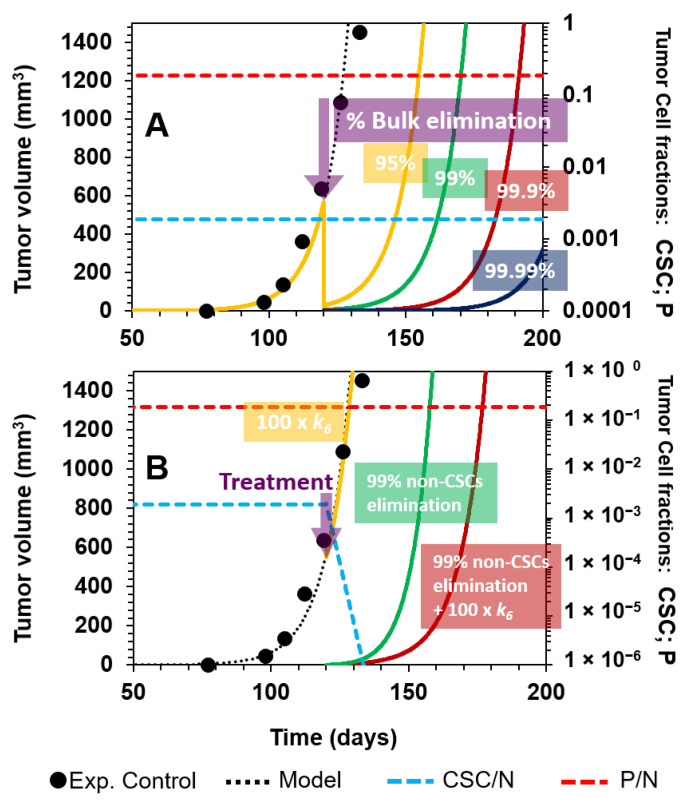
Nonselective treatment and directed therapy against CSCs. (**A**) Progression of a tumor without intervention is depicted from day 0 to 120 (black points, experimental data; dotted black curve, model fitting). At day 120 (purple arrow), the bulk of the tumor, CSCs, and P and D cells, is nonselectively eradicated: 95% (yellow line), 99% (green line), 99.9% (dark red line), and 99.99% (dark blue line). The CSC fraction (dashed blue line) and P fraction (dashed red line) remain unaltered for all nonselective treatments. (**B**) Selective targeting of CSCs at day 120: 100-fold enhancement of *k_6_* does not affect tumor progression (yellow line), although the fraction of CSCs diminishes (dashed blue line). When 99% of all cells except CSCs are eradicated at day 120 (green line), in addition to a 100-fold enhancement of *k*_6_, there is a better response (dark red line) than using either treatment alone.

We first tested the effect of a hypothetical nonselective treatment, such that 95%, 99%, 99.9%, or 99.99% of solid tumor cells were eradicated at day 120 (Figure 4A).

We modeled this hypothetical scenario by increasing the number of dead cells for each of the compartments of CSCs, P, and D cells without selective elimination, i.e., the indicated percentage of cells were eliminated as a bulk, assuming that dead cells were reabsorbed immediately. This is the reason why the tumor volume dropped abruptly at the time of intervention (day 120, indicated by the purple arrow in Figure 4A). After having performed this hypothetical treatment, the tumor relapsed in all cases, and the best scenario was a lag period of 60 days corresponding to the 99.99% elimination of total cells. Furthermore, the initial proportions of the different cell subpopulations were maintained (Figure 4A, dashed lines). This approach reflects the failure of some treatments, which do not eliminate the disease, despite destroying the bulk of the tumor.

#### 3.2.2. Selective Targeting of Less Differentiated Tumor Cells

We then explored the selective killing of CSCs. Our simulations showed that this therapeutic approach would not work better than the nonselective treatment discussed previously (Figure 4B). Using the same base case of Figure 4A, we applied a therapy in which, at day 120, CSCs are induced to die by a 100-fold augmentation of *k*_6_*,* representing the CSC-specific death rate in the cellular reaction R6 (see the Mathematical Model section). Although the fraction of CSCs is diminished, the tumor continues to grow at the same rate as in the absence of treatment (Figure 4B, yellow line). On the other hand, when 99% of all cells except CSCs are killed at day 120, the tumor relapses at day 140 (Figure 4B, green line). However, when this last strategy is combined with a 100× enhancement of *k*_6_, there is a better response than when using these approaches separately (Figure 4B, dark red line).

This last observation suggested that when the number of progenitors is too high, there is almost no effect when targeting only CSCs. However, when the number of P cells is reduced, there is a clear effect on the tumor volume when selectively inducing CSC death. This suggests that the selective killing of CSCs might be useful during tumorigenesis, when CSCs proliferate to give rise to tumors and their observed heterogeneity.

**Figure 5:**
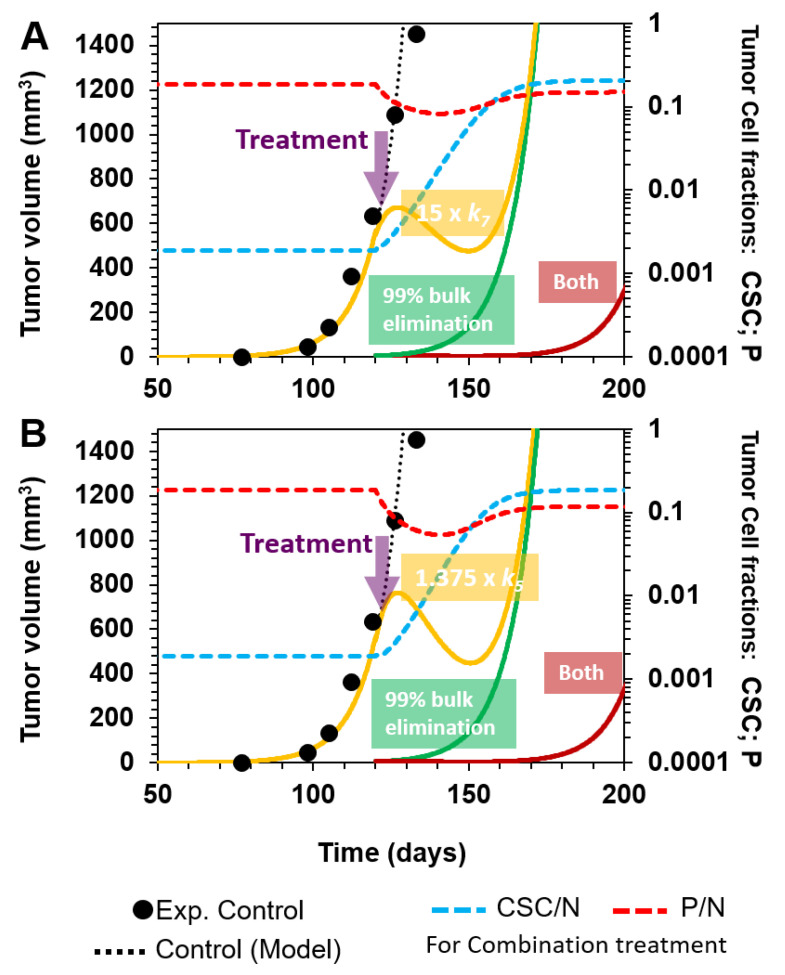
Targeting of intermediate P cells. (**A**) Selective elimination of P cells by increasing *k*_7_ 15-fold at day 120 (yellow line). This effect is comparable to the nonselective eradication of 99% of total tumor cells (green line). However, when both treatments are performed simultaneously, there is an increment of time before tumor relapse (dark red line). All treatments were started at day 120 (purple arrow). (**B**) Increasing P cell differentiation (Φ_5/4_ is increased from 0.8 to 1.1, which is equivalent to increasing 37.5% *k_5_*, at day 120; yellow line), eliminating 99% of total cells (green line), and the combination of both treatments (dark red line) have similar effects, as depicted in (**A**). The CSC fraction (dashed blue lines) and P cell fraction (dashed red lines) are shown for combination treatments in (**A**,**B**).

Following this, we examined strategies targeting P cells. First, we explored the selective killing of P cells by augmenting *k*_7_ 15-fold (P cell death rate in R7 in the cellular model). We observed that this therapeutic approach was as effective as a 99% eradication of total cells in terms of the tumor volume, with relapse occurring at day 140 (Figure 5A, yellow and green lines, respectively). Moreover, when we combined both treatments, the tumor relapse was delayed to day 180 (Figure 5A, dark red line), indicating a 3-fold improvement compared to the use of either individual treatment.

Another strategy would involve augmenting the relative intrinsic rate of progenitor cell differentiation (R5) with respect to the rate of progenitor cell renewal (R4), i.e., increasing Φ_5/4_. Interestingly, a 37.5% increase in Φ_5/4_ (from 0.8 to 1.1), while keeping the remaining Φ_i/j_ values fixed, retards the relapse of the tumor by approximately 20 days (Figure 5B, yellow line), similar to the nonselective elimination of 99% of total tumor cells (Figure 5B, green line) and the selective 15-fold increase in the P-cell specific death rate (Figure 5A, yellow line). Moreover, the combination of both treatments, involving the selective differentiation of P cells and nonselective 99% elimination of bulk tumor cells, slowed down tumor relapse by approximately 60 days (Figure 5B, dark red line).

In conclusion, both combination strategies, in which P cells and bulk tumor cells are targeted, have similar effects, with a 3-fold improvement compared to using each approach separately. This result suggests that P cells have the ability to maintain tumor growth. However, the impact of P cell targeting on the tumor volume depends on the fraction of P cells within the tumor. In addition, while an improvement in tumor volume reduction is observed, the fraction of CSCs is considerably increased (Figure 5 A,B, dashed blue lines), which would be a threatening event due to the intrinsic properties of CSCs.

#### 3.2.3. Combinatorial Targeting of Less Differentiated Tumor Cells

**Figure 6:**
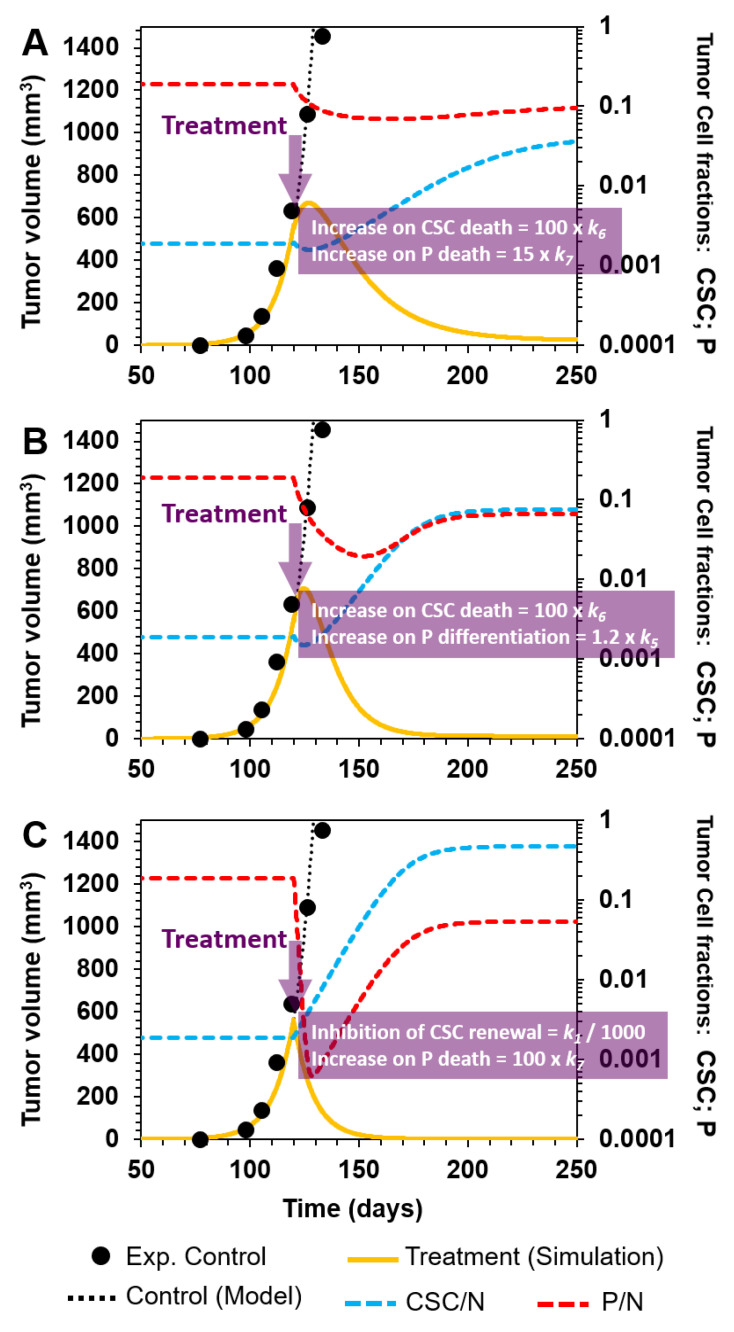
Combination therapy against CSCs and P cells. Yellow curves represent simulations of the simultaneous targeting of CSCs and P cells at day 120 (purple arrow). Scenario (**A**): Selective killing of CSCs and P cells by a 100- and 15-fold augmentation of their corresponding death rate kinetic parameters, *k*_6_ and *k*_7_, respectively. Scenario (**B**): Enhancement of P cell differentiation into D cells by increasing *k*_5_ by 20% and the selective elimination of CSCs by a 100-fold augmentation of *k*_6_. Scenario (**C**): Inhibition of symmetrical CSC renewal by a 1000-fold reduction in *k*_1_ and selectively killing P cells by increasing *k*_7_ 100-fold. For all cases: Experimental control data are represented by black points; model curve fit to experimental control data by the dotted black line; model curve for treatment simulation by solid yellow lines; and P and CSC tumor fractions by dashed red and blue lines, respectively.

Although targeting P cells at an advanced stage of tumor development has the potential to delay tumor growth, neither strategy explored above led to eradication of the tumor. However, simulations of several combinations showed that to effectively stop tumor growth in the time window applicable to our model, both CSCs and P cells must be targeted. For example, we simulated the following scenarios with therapy starting at day 120:

(A) Selective killing of CSCs and P cells by a 100- and 15-fold augmentation of *k*_6_ and *k*_7_, and their corresponding death rates in cellular reactions R6 and R7, respectively (Figure 6A);
(B) Enhancement of P cell differentiation into D cells by increasing *k*_5_ by 20% in cellular reaction R5, and selectively eliminating CSCs by augmenting *k*_6_ 100-fold in R6 (Figure 6B);
(C) Inhibition of CSC symmetrical renewal by a 1000-fold reduction in *k*_1_ (R1) and selectively killing P cells by a 100-fold increase in *k*_7_ (R7) (Figure 6C).

As shown in Figure 6A–C, these strategies have a substantial effect on the tumor burden. Apparently, the tumor is eliminated when using either combination strategy. However, the following question arises: How long must therapy be maintained so that there is no tumor relapse? In fact, it can be observed from the model output that even when using the most aggressive of the three scenarios described above (scenario C) in terms of tumor volume reduction (compare, for example, tumor volumes at day 150), there is still a fraction of CSCs and P cells at day 250 (Figure 6C). Importantly, the fraction of P cells diminished and that of CSCs augmented (Figure 6C, red and blue dashed lines, respectively), which suggests that the inhibition of CSC symmetrical renewal combined with bulk therapy may improve and control the tumor burden only temporally.

**Figure 7:**
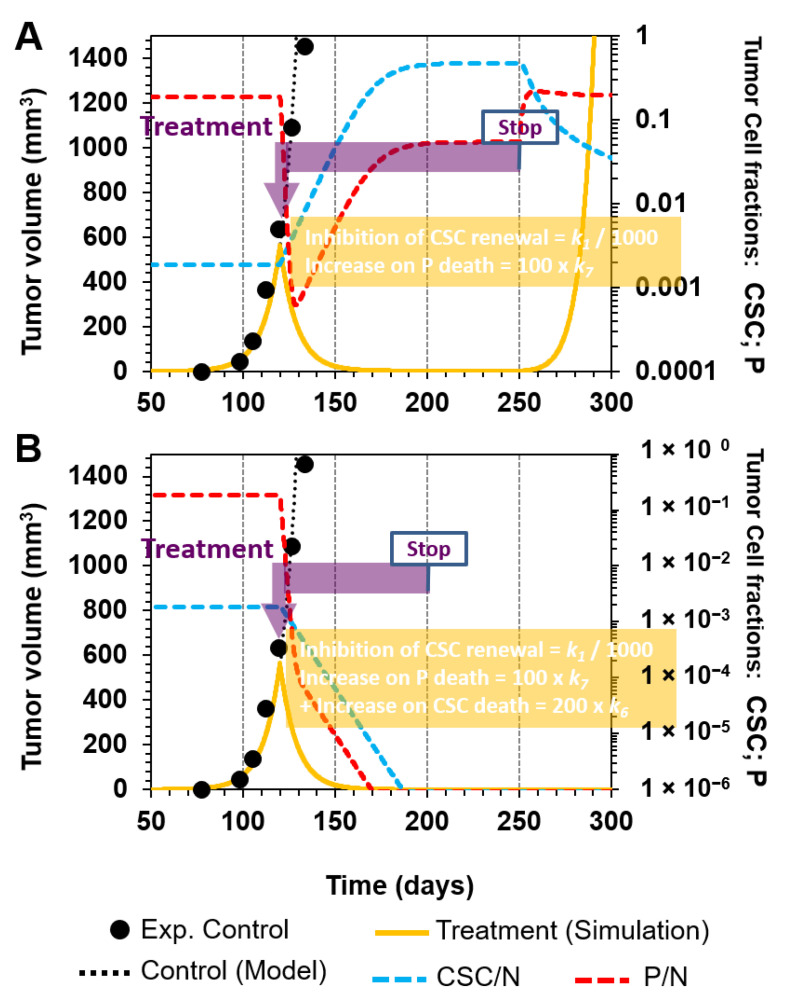
Time lapse for combination treatment. (**A**) When the treatment in Figure 6C (inhibition of symmetrical CSC renewal by a 1000-fold reduction in *k*_1_ and selectively killing P cells by increasing *k*_7_ 100-fold) was selected and stopped at day 250, there was a tumor relapse at day 280 due to residual tumorigenic cells that were not eliminated when the treatment was stopped (see CSC and P cell fractions). (**B**) Selectively killing CSCs by increasing *k*_6_ 200-fold in addition to the treatment in (**A**) eradicated the tumor, which did not relapse, even when therapy was stopped at day 200. P and CSC tumor fractions are represented by dashed red and blue lines, respectively.

In this regard, we simulated a scenario in which the combination therapy (C) was stopped at day 250, 130 days after starting the treatment, when observing that the disease was eliminated (Figure 7A, yellow line; treatment time is highlighted in purple). Since a fraction of target cells were still present when the treatment was stopped, the resulting effect was that the tumor relapsed in approximately 40 days after stopping the treatment and continued its exponential growth (Figure 7A, yellow line).

Therefore, we next evaluated a more aggressive therapy in which, in addition to the combination treatment in scenario C, there was a 200-fold increase in *k_6_*, which is the rate constant of CSC death (R6). In this case, the model output indicated no tumor relapse, even when the therapy was stopped at day 200, due to the elimination of all CSCs and P cells before that point, as can be observed in the fraction curves (Figure 7B). As our model suggests, it is important not only to simultaneously target CSCs and P cells, but also to do so for a sufficiently long enough period of time, in order to ensure their effective and complete elimination.

### 3.3. Further Methods and Models to Verify the Hypothesis

#### 3.3.1. Selective Targeting of CSCs in Combination with Standard-of-Care Chemotherapy in Ovarian Cancer

Ovarian cancer (OvCa) is the most lethal gynecological cancer in the United States [49]. According to the American Cancer Society, 21,750 women will receive a new diagnosis and 13,940 women will die from ovarian cancer in 2020. Typical treatment for OvCa includes surgical resection, radiotherapy, and chemotherapy [49].

First-line chemotherapy based on platinum and taxane agents allows a clinical response, but the rate of recurrence is high, and the survival rate from recurrent disease is poor due to more resistant cancer cells. It has been demonstrated that OvCa stem cells (OvCSCs) are more resistant to this treatment and, hence, they have been proposed as targets for therapy [47].

**Figure 8:**
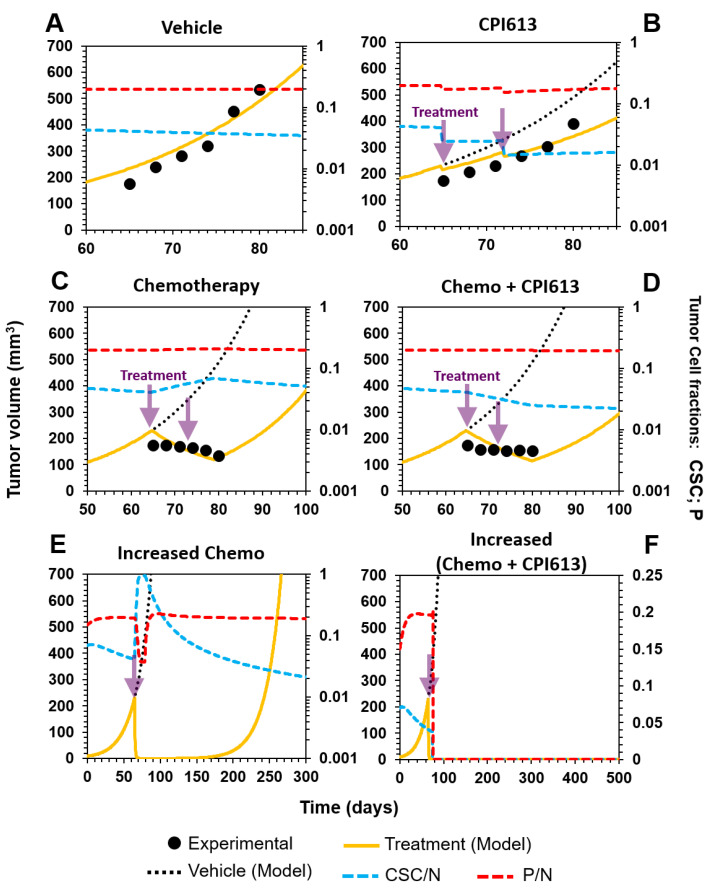
Combination treatment of ovarian cancer. (**A**) Control treatment with a vehicle. (**B**) Selective targeting of CSCs by treatment with CPI613. (**C**) Chemotherapy based on carboplatin and paclitaxel. (**D**) Combination therapy (chemotherapy + selective targeting of CSCs). (**E**) Augmentation of the chemotherapy dose retarded tumor relapse, but did not eliminate the disease. (**F**) Treatment in (**E**), combined with an increased dose of CPI613, produced a better response than either treatment alone. Furthermore, tumor relapse was not observed. For all cases: Experimental data are represented by black points; model simulation by solid yellow lines; control treatment by a dotted black line; and P and CSC tumor fractions by dashed red and blue lines, respectively. The treatment time is represented with purple arrows.

Indeed, Bellio et al. [47] demonstrated that after treating mice with induced OvCa with carboplatin and paclitaxel, delayed tumor growth was observed, concomitant with an enrichment in the CSC fraction. We simulated this treatment in our model by changing the original numbers of cells and corresponding cell fractions, and by increasing bulk tumor cell death. We observed that the experimentally observed tumor curve and enrichment in CSCs from 3% (Figure 8A) to 5% were also reproduced by our model, as shown in Figure 8C.

Interestingly, Bellio and coworkers showed that after selectively targeting OvCSCs with CPI613, which is a carboxylic acid cycle inhibitor, reductions in the tumor volume and CSC fraction were observed after two weeks of treatment. Again, our model also showed this observed delay in the tumor curve when only targeting CSCs. In addition, the model reproduced the reduction in the CSC fraction from 7.65% in the inoculation culture to 1.64%, as shown experimentally (Figure 8B).

Finally, the authors explored a combination therapy using chemotherapy and CPI613 administration with the hope of improving tumor treatment. This resulted in a retarding effect on tumor growth that was equivalent to that observed for chemotherapy treatment alone (compare Figure 8C,D). Despite there being no improvement in the overall tumor volume growth compared to chemotherapy alone during the assessed time frame, the CSC fraction was reduced with respect to the individual carboplatin–paclitaxel treatment (Figure 8C vs. Figure 8D).

We simulated this combination therapy and not only observed the same experimental behavior in the time window of the experiment, but also predicted tumor relapse past the last experimental data point (Figure 8D). We then thought of a more aggressive combination treatment by increasing the CSC and P cell death rates. We show that the tumor is eradicated using this strategy (Figure 8F) and that no relapse is observed compared to more aggressive nonselective treatment, with an increase in only the dose of carboplatin–paclitaxel (Figure 8E). This result corresponds with what we discussed in the previous section, suggesting that, for effective treatment, both CSC and P compartments should be targeted in a modality that ensures their eradication.

#### 3.3.2. Sketching an In Vivo Experiment

We propose an experiment in which tumors in mice can be completely visualized. The success of this model will rely on the ability to distinguish and track CSCs, as well as P and D cells. The proposed therapies can be evaluated and tumor progression observed. Engineered cells might be implemented such that they can be traced during tumor progression using a fluorescent protein that is reported, depending on the expression of a gene of interest [27], and/or a specific subpopulation becomes sensitive to an agent such as a toxin [28,29]. Single-cell analysis and DNA-barcoding tracking would also complement the experiment [16,22].

### 3.4. Model Limitations

Seeing tumors as single malignant entities may limit our understanding of the failure of some cancer therapies. Tumor heterogeneity and the tumor microenvironment play important roles in cancer development [1]. The recent imaging of tumors as ecosystems consisting of supporting cell niches, tumor vessels, cancer stem cell niches, and immune cells represents a step forward [50,51] and may assist with attempts to gain further insight into the processes of cancer progression and development of resistance to therapy. Furthermore, from a genetics perspective, cancers include genetic aberrations that could eventually lead to additional mutations and/or the expression of genes under selective pressure, such as from a specific drug, which could be reflected as resistance to treatment.

The model that we present here does not attempt to evaluate the contribution of each tumor ecosystem component or the influence of the mutational rate and/or selective pleiotropy in tumor cells. Instead, the model focuses on the structure of a unidirectional lineage of cancer stem cells giving rise to different tumor cell subpopulations. This simplistic approach bypasses the array of possibilities that could lead to cancer resistance, for example, the effect of an additional mutation in a clone, the plasticity of cancer cells, the possible presence of different CSCs and their own hierarchies within the same tumor, or the contribution of a supporting cell niche to cancer cell survival.

Here, the presented model is only descriptive of the overall exponential primary tumor growth. Indeed, many aspects of tumor biology are not punctually considered. For example, recent evidence suggests that CSCs can be quiescent, which would imply that they do not proliferate during a time lapse of tumor development [7,27]. Our model does not specifically address this transient behavior. Instead, the parameter *k* might be considered as an average of the kinetic rate constants that mediate a particular cellular event “R”. For example, if CSCs are quiescent during a certain period, then *k_1_* = 0 during that time; but if they resume proliferation later at a rate of *k_1_* = 1, then the average *k_1_* would be 0.5. Notwithstanding this important remark, the rates of cellular divisions where CSCs are involved are of a relative lower value compared to P cells, i.e., Φ_4/1_ = 5.35 and Φ_5/4_ = 0.8, which is consistent with the slower average proliferation rate of CSCs.

Additionally, the model does not consider the plasticity of cancer cells. We departed from the principles that this phenomenon has been mostly observed in the metastatic process [19,29], that the deletion of EMT did not affect primary tumor growth in a mouse model [21], and that an invariable stem cell hierarchy was observed in glioblastoma tumors [22]. Although the inclusion of plasticity would represent a more complete scenario, we retained a unidirectional lineage of a stem cell hierarchy to keep the modeling frame simple, according to the cancer stem cell hypothesis. When framed in this way, we could fit the model to the available experimental data. Modeling plasticity may help understand other aspects of tumor dynamics or hypothesize additional therapeutic strategies. However, as the complexity is increased, a more sophisticated model needs to be considered that could be linked to experimental input data related to the spatial distribution of specific features driving cell plasticity, e.g., signals from a specific niche.

Finally, this model does not consider the invasion behavior that different cell subpopulations could have. Again, it is descriptive of the overall tumor growth, and many aspects are concentrated in the calculated average density. An improved version of this model would link the cellular events (R) and their specific kinetic parameters (*k*) with a systems biology part. Both parts could be connected in a loop for the feedback input. Such a hybrid model could help understand or reveal molecular features of tumor growth. Here, we did not aim to construct such a model. Our objective was to fit a minimal model descriptive of the overall dynamics of primary tumor growth and in line with the cancer stem cell hypothesis, in order to aid in the visualization of the tumor response when different subpopulations are targeted individually or in combination. This type of model, although simplistic, may help to follow the average behavior of tumor growth in certain types of tumors, such as glioblastoma, and get a picture of potential treatment strategies against tumor growth. Then, the observed theoretical output can be tested experimentally and compared with experimental results. Based on this, the model can be redefined or its parameters can be refined.

## 4. Concluding Remarks

Because of the recurrence of tumors after conventional treatment, there have been ongoing efforts to develop selective therapies against CSCs. However, experimental data reported in the literature demonstrate that this approach is not totally efficient. We propose that a combination therapy targeting both CSCs and progenitor (P) cells displaying intermediate characteristics between poorly differentiated and mature cells would represent a better treatment for tumor eradication. We tested this hypothesis using a simple mathematical model that was adjusted to the experimental data considering selective CSC targeting. We further evaluated therapies ranging from nonselective to specifically directed and combination therapy, and showed that CSC targeting is useful both during carcinogenesis, when few P cells are present, and in combination with P cell targeting when the tumor burden contains a significant amount of P cells.

Although our approach is theoretical, there is recent evidence of an invariant stem cell hierarchy in glioblastoma tumors giving rise to progenitor cells with an extensive self-maintenance capacity [22,31]. There is also evidence of subpopulations of cells showing a differential expression of embryonic stem cell markers in head and neck squamous cell carcinoma, colon adenocarcinoma, melanoma, and buccal squamous cell carcinoma [23,33,34,35], in addition to evidence that more differentiated cells can generate tumors [9,10,17,25,28]. Moreover, recent findings on antibody-drug conjugates (ADC) showed that the targeting of Lgr5(+) colon CSCs with an ADC controlled the disease, but did not eliminate it, as observed in their reported growth curves [52]. Interestingly, in another study on head and neck squamous cell carcinoma, using an ADC with a wider spectrum showing toxicity to both CSCs and non-CSCs eliminated the disease, with no relapse occurring during the evaluation period [53]. Therefore, testing the proposed hypothesis could at least allow us to discriminate among the various possibilities of tumor resistance to cancer therapy. The results may also encourage researchers to see the different subpopulations within the tumor as a potential risk for the tumor burden, and thus think about a combinatorial strategy, rather than only focusing on CSCs and leaving bulk tumor cells without intervention. In the future, personalized combination therapy against different cell subpopulations may become possible, and for that reason, single-cell analyses of tumors would be necessary as companion diagnostics, as suggested elsewhere [16].

## Figures and Tables

**Table 1:** Summary of the rates of consumption and production of CSCs.

Reaction	$r_{production,CSCs}$	$r_{cell\;division,CSCs}$	$r_{cell\;death,CSCs}$
R1	$2k_{1}CSC$	$k_{1}CSC$	0
R2	$k_{2}CSC$	$k_{2}CSC$	0
R3	0	$k_{3}CSC$	0
R6	0	0	$k_{6}CSC$
Total	$2k_{1}CSC+k_{2}CSC$	$k_{1}CSC+k_{2}CSC+k_{3}CSC$	$k_{6}CSC$
